# Poor Long-Term Outcome in Second Kidney Transplantation: A Delayed Event

**DOI:** 10.1371/journal.pone.0047915

**Published:** 2012-10-23

**Authors:** Katy Trébern-Launay, Yohann Foucher, Magali Giral, Christophe Legendre, Henri Kreis, Michèle Kessler, Marc Ladrière, Nassim Kamar, Lionel Rostaing, Valérie Garrigue, Georges Mourad, Emmanuel Morelon, Jean-Paul Soulillou, Jacques Dantal

**Affiliations:** 1 Institut de Transplantation Urologie Néphrologie (ITUN), Inserm U643, CHU Hôtel Dieu, Nantes, France; 2 Université de Nantes, EA4275 ‘Biostatistique, Recherche Clinique et Mesures Subjectives en Santé’, Nantes, France; 3 Service de Transplantation Rénale et de Soins Intensifs, Hôpital Necker, APHP, Paris, France; 4 Universités Paris Descartes et Sorbonne Paris Cité, Paris, France; 5 Service de Transplantation Rénale, CHU Brabois, Nancy, France; 6 Service de Néphrologie, HTA, Dialyse et Transplantation d'Organes, CHU Rangueil, Toulouse, France; 7 Université Paul Sabatier, Toulouse, France; 8 Service de Néphrologie-Transplantation, Hôpital Lapeyronie, Montpellier, France; 9 Service de Néphrologie, Transplantation et Immunologie Clinique, Hôpital Edouard Herriot, Lyon, France; University of Colorado Denver, United States of America

## Abstract

**Background:**

Old studies reported a worse outcome for second transplant recipient (STR) than for first transplant recipient (FTR) mainly due to non-comparable populations with numbers confounding factors. More recent analysis, based on improved methodology by using multivariate regressions, challenged this generally accepted idea: the poor prognosis for STR is still under debate.

**Methodology:**

To assess the long-term patient-and-graft survival of STR compared to FTR, we performed an observational study based on the French DIVAT prospective cohort between 1996 and 2010 (N = 3103 including 641 STR). All patients were treated with a CNI, an mTOR inhibitor or belatacept in addition to steroids and mycophenolate mofetil for maintenance therapy. Patient-and-graft survival and acute rejection episode (ARE) were analyzed using Cox models adjusted for all potential confounding factors such as pre-transplant anti-HLA immunization.

**Results:**

We showed that STR have a higher risk of graft failure than FTR (HR = 2.18, p = 0.0013) but that this excess risk was observed after few years of transplantation. There was no significant difference between STR and FTR in the occurrence of either overall ARE (HR = 1.01, p = 0.9675) or steroid-resistant ARE (HR = 1.27, p = 0.4087).

**Conclusions:**

The risk of graft failure following second transplantation remained consistently higher than that observed in first transplantation after adjusting for confounding factors. The rarely performed time-dependent statistical modeling may explain the heterogeneous conclusions of the literature concerning second transplantation outcomes. In clinical practice, physicians should not consider STR and FTR equally.

## Introduction

Nowadays, repeat transplantation provides the best chance for long-term survival and quality of life in patients facing allograft loss, as compared to maintenance dialysis therapy [1], [2], [3]. This concept was recently supported by Ojo et al. [2] who showed that repeat transplantation is associated with a reduced mortality compared to remaining on dialysis after a prior graft loss. This benefit is valid despite the fact that re-transplant recipients present a higher risk of death during the first month after the transplant surgery [1]. When considering short and long-term outcomes, graft survival rates following retransplantation have continuously improved in recent years [4]. There is evidence that patients undergoing a third or more transplantation have a worse prognosis [5], [6], [7]. However, the poor prognosis of second transplant recipients (STR) remains a matter of debate.

Some previous studies have demonstrated that STR have a lower graft survival than first transplant recipients (FTR) [2], [8], [9], [10], [11], [12] leading STR to be considered as a higher risk group for graft failure, mainly related to increased levels of preformed HLA antibodies [13]. However, Coupel et al. showed that the difference in long-term graft survival was not significant between STR and FTR when an HLA-DR mismatch was avoided [14]. Recent improvements in immunosuppressive therapy may have contributed to decreasing the difference in outcomes between STR and FTR [8]. When taking into account several confounding factors such as pre-transplant immunization, evidence of an excess risk of graft failure for STR is not clear, as demonstrated by the most recent studies [1], [5], [15], [16]. For Magee et al., after adjustment for donor and recipient factors, the risk of graft failure remained significantly higher for STR than FTR [17].

Whereas factors influencing second graft survival have been well studied [8], [9], [14], [16], [18], [19], [20], those related to a possible excess risk of graft failure for STR compared with FTR are not well established [15], [17]. The objective of our study was not to recommend whether patients should get a second transplant or not. Addressing this important question would require a completely different study design. Indeed, the overall aim of our epidemiological observational cohort study was to provide data from a large multicenter population of kidney transplant recipients in order to clarify the relationship between the graft rank and the long term graft outcomes. For the first time, we adjusted for a large number of covariates at baseline and we modeled the time-dependent relationship between graft rank and graft survival. According to these methodological improvements, we demonstrated that STR have a poorer patient-and-graft survival (PGS) than FTR significant since four years post-transplantation.

## Materials and Methods

### Study population

Data were prospectively collected from the DIVAT (Données Informatisées et VAlidées en Transplantation) French multicentric database [21]. Codes were used to assure donor and recipient anonymity and blind assay. The “Comité National Informatique et Liberté” approved the study (N° CNIL 891735) and written informed consent was obtained from the participants. The data are computerized in real time as well as at each transplant anniversary and are submitted for an annual audit. The cohort consisted of 2462 FTR and 641 STR meeting the following inclusion criteria: (a) adult recipients; (b) transplantations performed between January 1996 and November 2010; and (c) maintenance therapy with calcineurin inhibitors, mammalian target of rapamycin inhibitors or belatacept, in addition to mycophenolic acid (Myfortic® Novartis, France or Cellcept® Roche, France) and steroids. Simultaneous transplantations were excluded. Among 2462 FTR meeting the inclusion criteria, 52 patients were also part of the STR group as they received two transplants during the observation period. These 52 patients, who were included in both cohorts, represented 2% and 8% of the FTR and STR groups respectively. Given the large number of covariates, it is reasonable to assume conditional independence of these patients. We did not exclude these 52 patients as this would have reduced the comparability of the two groups by under-representing the FTR patients with a rapid return-to-dialysis, which would have led to an over-estimation of FTR graft survival.

### Clinical variables of interest

To guarantee the comparability between FTR and STR, adjustments were made for all of the following possible pre- or per-transplant immunological and non-immunological confounding factors: transplantation period (before or after 2005, which corresponds to the routine utilization of high sensitivity techniques for panel-reactive antibody, PRA), recipient gender and age, primary diagnosis of end stage renal disease (ESRD), comorbidities, highest historical levels of pre-transplant PRA against class I and II antigens, deceased or living donor status, donor age, cold ischemia time (CIT), HLA-A-B-DR mismatches and induction therapy. The high sensitivity techniques correspond to pre-transplant anti-HLA identification obtained by multiplex screening test (LAT-M; One lambda, Canoga Park, CA).

French law does not authorize the storage of race information (specific authorization may be obtained in specific circumstances, such as for genetics population studies). The induction therapy was differentiated according to its effect on lymphocytes: horse or rabbit antithymocyte globulin antibodies or anti-CD3 antibody were considered as lymphocyte-depleting agents whereas anti-interleukin-2 receptor antibodies (basiliximab) were considered as a non lymphocyte-depleting agent. Since not all of the biopsies were analyzed with the recent Banff classification but as therapeutic strategies were nevertheless mostly based on a histological diagnosis regardless of the time period, and as the therapeutic strategies did not differ according to the graft rank regardless of the period, we opted to grade acute rejection episode (ARE) according to their response to steroid bolus therapy: steroid-sensitive ARE were considered as non-severe, whereas steroid-resistant ARE requiring rescue with additional therapy were considered as severe.

### Statistical analysis

Comparisons of baseline characteristics between the FTR and STR were based on the chi-square test. Different times-to-event distributions were described including the time between the transplantation and: (a) the graft failure, i.e. the first event between the return to dialysis and the patient death with a functioning graft (patient-and-graft survival); (b) the return to dialysis, i.e. patient deaths were censored (graft survival); (c) the patient death with a functioning graft, i.e. the returns to dialysis were censored (patient survival); (d) the first ARE and (e) the first severe ARE, i.e. non-severe ARE were censored. Survival curves were estimated using the Kaplan-Meier estimator. Only the main outcomes, i.e. PGS and ARE/severe ARE occurrences, were analyzed in multivariate. A first selection of covariates using the Log-rank test (p<0.20) was performed before the Cox model (Wald test with p<0.05, step-by-step descending procedure). Cox models were stratified per center. Baseline parameters differentially distributed between FTR and STR were also introduced in the models. Because the Cox model was performed by using all the recipients regardless the graft rank, we did not take into account specific covariates for STR, such as the survival time of the first transplant [15], [18] or the time in dialysis before retransplantation [10], [15], [19]. Because the definition of the duration in ESRD is different between FTR and STR, a special attention was paid to ESRD-related comorbidities.

Hazards proportionality was checked by plotting log-minus-log survival curves and by testing the scaled Schoenfeld residuals [22]. Interactions between the graft rank and all the covariates were tested. The possible colinearity between donor type and CIT was also checked. An extended Cox model with time-dependent coefficients was used for non-proportional covariates [23], [24]. The change time-point of the hazard ratio was estimated by minimizing the Bayesian Information Criteria [25]. In order to evaluate graft survival for the two comparable populations of FTR and STR, we also performed a sub-analysis. According to the independent risk factors for graft failure highlighted by the previous methodology, we identified 486 pairs of FTR and STR. A Kaplan-Meier estimator and the Cox model were also used to evaluate the association between graft rank and graft survival in this sub-analysis.

Statistical analyses were performed using version 2.12.0 of the R software [26].

## Results

### Description of the cohort

The demographic and baseline characteristics at the time of transplantation are presented in Table 1. Among the 3103 kidney transplantations, 641 (20.7%) were STR. In both groups, the majority of patients received a transplant from a deceased donor, after a period of dialysis, and the distributions of recipient and donor gender were comparable. STR were younger (p<0.0001) and their transplants came from younger donors (p<0.0001). Recurrent nephropathies (p<0.0001), cardiac disease (p = 0.0007), hepatitis (p<0.0001) and malignancy (p<0.0001) were more frequent among STR. Compared to FTR, STR received better HLA-matched transplants (p<0.0001), but their CIT were longer (p<0.0001) and they were more sensitized, with higher positivity of anti-class I and anti-class II PRA than FTR (p<0.0001). They were also more frequently exposed to induction therapy with a lymphocyte-depleting agent (p<0.0001).

**Table 1:** Demographic and baseline characteristics of primary and second transplants performed in the DIVAT network between January 1996 and November 2010.

Characteristics	All grafts (N = 3103)		First graft (N = 2462)		Second graft (N = 641)	
	N	(%)	N	(%)	N	(%)
Transplantation period<2005	887	(28.6%)	685	(27.8%)	202	(31.5%)
Male recipient	1900	(61.2%)	1516	(61.6%)	384	(59.9%)
Recipient≥55 years of age	1295	(41.7%)	1100	(44.7%)	195	(30.4%)*
Potentially recurrent causal nephropathy	1016	(32.7%)	744	(30.2%)	272	(42.4%)*
Presence of dialysis prior transplantation	2782	(89.8%)	2197	(89.5%)	585	(91.3%)
History of diabetes	336	(10.8%)	295	(12.0%)	41	(6.4%)*
History of hypertension	2527	(81.4%)	2013	(81.8%)	514	(80.2%)
History of vascular disease	381	(12.3%)	296	(12.0%)	85	(13.3%)
History of cardiac disease	1011	(32.6%)	766	(31.1%)	245	(38.2%)*
History of dyslipemia	880	(28.4%)	731	(29.7%)	149	(23.2%)*
History of malignancy	248	(8.0%)	161	(6.5%)	87	(13.6%)*
History of B or C hepatitis	191	(6.2%)	110	(4.5%)	81	(12.6%)*
Positive recipient CMV serology	1844	(59.8%)	1413	(57.8%)	431	(67.6%)*
Positive recipient EBV serology	2886	(96.1%)	2289	(96.0%)	597	(96.8%)
Recipient BMI≥30 kg.m^−2^	291	(9.5%)	256	(10.5%)	35	(5.5%)*
Positive anti-class I PRA	822	(26.5%)	420	(17.1%)	402	(62.7%)*
Positive anti-class II PRA	889	(28.6%)	410	(16.7%)	479	(74.7%)*
Male donor	1817	(58.9%)	1421	(58.0%)	396	(62.4%)*
Donor≥55 years of age	1265	(40.8%)	1056	(42.9%)	209	(32.6%)*
Deceased donor	2785	(89.8%)	2181	(88.6%)	604	(94.2%)*
Cerebro-vascular cause of donor death	1480	(49.7%)	1168	(49.7%)	312	(50.0%)
Donor serum creatinine≥133 µmol/l	385	(12.6%)	311	(12.9%)	74	(11.8%)
Positive donor CMV serology	1582	(51.2%)	1266	(51.6%)	316	(49.7%)
Positive donor EBV serology	2639	(94.3%)	2104	(94.6%)	535	(93.0%)
HLA-A-B-DR incompatibilities>4	432	(13.9%)	390	(15.8%)	42	(6.6%)*
HLA-A incompatibilities≥1	2437	(78.5%)	1993	(81.0%)	444	(69.3%)*
HLA-B incompatibilities≥1	2774	(89.4%)	2248	(91.3%)	526	(82.1%)*
HLA-DR incompatibilities≥1	2310	(74.4%)	1919	(77.9%)	391	(61.0%)*
Cold ischemia time≥24 h	905	(29.2%)	668	(27.1%)	237	(37.0%)*
Induction with a lymphocyte-depleting agent	1385	(44.7%)	883	(35.9%)	502	(78.3%)*

BMI, body mass index; PRA, panel reactive antibody; HLA, human leukocyte antigen; CMV, cytomegalovirus; EBV, Epstein-Barr virus.

* p<0.05.

### Survival analysis

**Figure 1:**
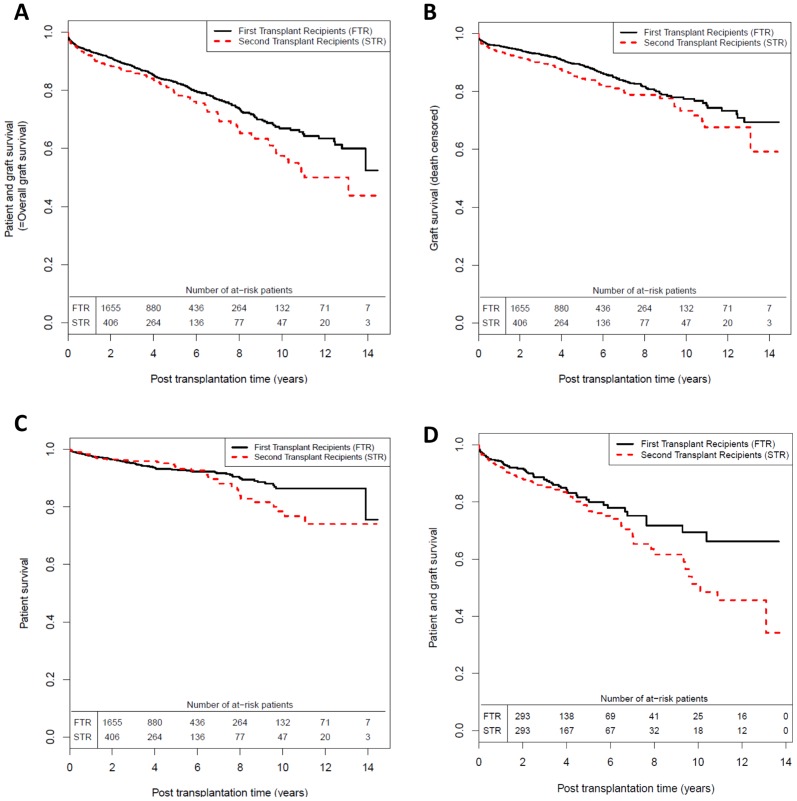
Unadjusted patient and/or graft survival analysis. (**A**) Patient-and-graft survival ( = overall graft survival) : patient deaths with a functioning graft are considered as a graft failure (log-rank test: p = 0.0127) (**B**) Death-censored graft survival: patient deaths with a functioning graft are censored (log-rank test: p = 0.0206) and (**C**) Patient survival: returns to dialysis are censored (log-rank test: p = 0.2890), for first and second grafts performed in the DIVAT network between January 1996 and November 2010 (Kaplan-Meier estimates). (**D**) Patient-and-graft survival sub-analysis for a sample of matched first grafts (N = 486) and second grafts (N = 486) for the following risk factors of graft failure: transplantation period, recipient age, history of cardiac disease, anti-class I PRA, recipient/donor relationship, BMI and EBV serology.

The patient-and-graft survival at 1, 5 and 10 years respectively were: 92%, 79% and 56% for STR and 94%, 83% and 66% for FTR (Figure 1-A). Without any adjustment on confounding factors, STR had a significantly higher risk of graft failure than FTR (p = 0.0127). Approximately beyond 4 years post-transplantation, the difference in survival curves appeared to increase over time. STR also had a significantly lower graft survival than FTR (Figure 1-B, p = 0.0206). However, we could not demonstrate a significant difference between the patient survival (Figure 1-C, p = 0.2890).

*The univariate analysis* revealed that the relationship between the graft rank and the PGS changed with post transplantation time (p = 0.0125). Assuming that the hazard ratio (HR) associated with the graft rank can be considered constant within the 2 periods; we found that the optimal cutoff point which minimized the Bayesian Information Criterion was 4 years. This model was validated by the analysis of the Schoenfeld's residuals. It was also coherent with Figure 1-A. Of note, graft failure was not significantly associated with the HLA-A-B-DR level (HR = 1.14, p = 0.274) nor with the HLA-DR level (HR = 1.03, p = 0.739).

*The multivariate analysis* was based on 2772 patients, as 257 FTR and 74 STR presented missing data (Table 2). The risk of graft failure was 2.18 times higher for STR after 4 years of transplantation (p = 0.0013). There was no significant difference before 4 years (HR = 1.05, p = 0.7830). The risk of graft failure was also higher for transplantation before 2005 (HR = 1.32, p = 0.0427), recipient≥55 years of age (HR = 1.49, p = 0.0012), deceased donor (HR = 2.19, p = 0.0015), cardiac disease (HR = 1.34, p = 0.0057), positive anti-class I PRA (HR = 1.43, p = 0.0055), obesity (HR = 1.54, p = 0.0050) and positive donor EBV serology (HR = 1.80, p = 0.0076). Of note, no interaction with the graft rank achieved statistical significance.

**Table 2:** Multivariate Cox model analysis of graft failure risk factors (N = 2772, as 257 first transplant recipients and 74 second transplant recipients presenting missing data for one of the covariates were deleted).

Variables	Hazard Ratio	95% CI	p
Graft rank before 4 post-transplant years (2/1)	1.05	0.75–1.47	0.7830
Graft rank after 4 post-transplant years (2/1)	2.18	1.35–3.50	0.0013
Transplantation period (<2005/≥2005)	1.32	1.01–1.72	0.0427
Recipient gender (male/female)	1.01	0.82–1.25	0.9364
Recipient age (≥55 years/<55 years)	1.49	1.17–1.89	0.0012
Causal nephropathy (recurrent/non recurrent)	1.13	0.91–1.39	0.2734
History of diabetes (positive/negative)	1.28	0.96–1.71	0.0947
History of hypertension (positive/negative)	0.86	0.67–1.12	0.2665
History of vascular disease (positive/negative)	1.05	0.80–1.38	0.7449
History of cardiac disease (positive/negative)	1.34	1.09–1.65	0.0057
History of dyslipemia (positive/negative)	1.16	0.93–1.45	0.1971
History of malignancy (positive/negative)	1.17	0.84–1.62	0.3483
History of B/C hepatitis (positive/negative)	1.06	0.72–1.57	0.7587
Number of HLA-A-B-DR mismatches (>4/≤4)	1.30	0.99–1.71	0.0639
Anti-class I PRA (positive/negative)	1.43	1.11–1.85	0.0055
Anti-class II PRA (positive/negative)	0.98	0.74–1.30	0.8970
Induction therapy (depleting/non depleting)	0.88	0.69–1.12	0.2852
Cold ischemia time (≥24 h/<24 h)	1.18	0.95–1.45	0.1370
Donor age (≥55 years/<55 years)	1.19	0.94–1.49	0.1459
Recipient/donor relationship (deceased donor/living donor)	2.19	1.35–3.57	0.0015
BMI (≥30 kg.m^−2^/<30 kg.m^−2^)	1.54	1.14–2.09	0.0050
Donor EBV serology (positive/negative)	1.80	1.17–2.77	0.0076

CI, confidence interval; BMI, body mass index; PRA, panel reactive antibody; HLA, human leukocyte antigen; EBV, Epstein-Barr virus.

*The sub-analysis* consisted of analyzing 486 pairs of FTR and STR with the same risk factors of graft failure (transplantation period, recipient age, history of cardiac disease, anti-class I PRA, recipient/donor relationship, BMI and EBV serology). The corresponding graft survivals are presented in Figure 1D. This confirmed the time-dependent effect of the graft rank: the risk of graft failure was 2.15 times higher for STR after 4 years of transplantation (95% CI = 1.14–4.08, p = 0.0184) but there was no significant difference before 4 years (HR = 1.11, 95% CI = 0.77–1.58, p = 0.5842).

### Acute rejection episode analysis

In order to explain the previous delayed excess risk in the STR group after few years post transplantation, we first made the hypothesis of a higher frequency of ARE or severe ARE in this group, with the associated delayed consequences on the patient-and-graft survival.

**Figure 2:**
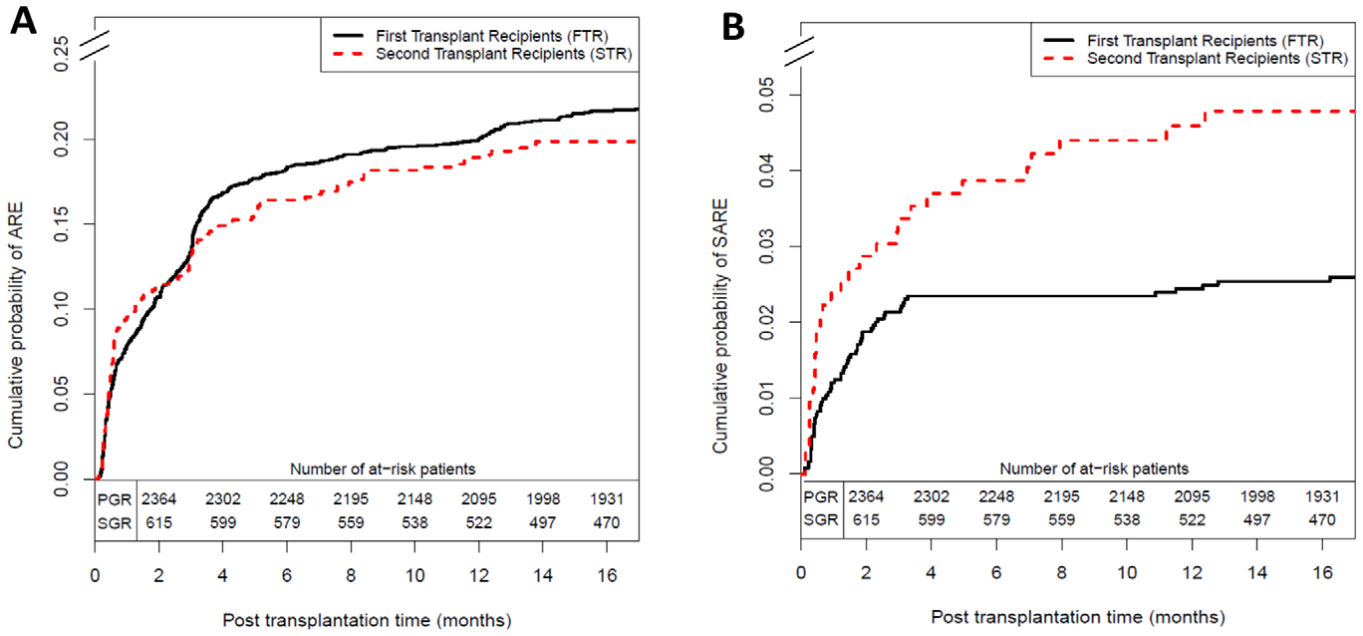
Cumulative probability of acute rejection episodes. (**A**) Cumulative probability of acute rejection episodes for FTR and STR (log-rank test: p = 0.4420) and (**B**) Cumulative probability of severe acute rejection episodes for FTR and STR (log-rank test: p = 0.0040), for first and second grafts (Kaplan-Meier estimates).

The cumulative probability of ARE at 1, 3 and 12 months respectively were 10%, 13% and 19% for STR and 8%, 14% and 20% for FTR (Figure 2-A). The univariate analysis showed no trend for higher ARE occurrence in STR than FTR (p = 0.4420). The multivariate Cox model confirmed this result (Table 3, HR = 1.01, p = 0.9675). ARE occurrence was related to HLA-A-B-DR mismatches (HR = 1.46, p = 0.0004) and anti-class II PRA (HR = 1.29, p = 0.0180). Recipients≥55 years of age (HR = 0.79, p = 0.0173) and recipients with a lymphocyte-depleting therapy (HR = 0.65, p<0.0001) had a lower risk of ARE occurrence.

**Table 3:** Multivariate Cox model analysis of acute rejection episode (ARE)-free time risk factors (N = 3103).

Variables	Hazard Ratio	95% CI	p
Graft rank (2/1)	1.01	0.80–1.27	0.9675
Transplantation period (<2005/≥2005)	0.80	0.64–1.01	0.0592
Recipient gender (male/female)	1.13	0.97–1.33	0.1233
Recipient age (≥55 years/<55 years)	0.79	0.65–0.96	0.0173
Causal nephropathy (recurrent/non recurrent)	1.03	0.88–1.22	0.6928
History of diabetes (positive/negative)	1.17	0.92–1.48	0.2128
History of hypertension (positive/negative)	1.18	0.96–1.45	0.1127
History of vascular disease (positive/negative)	1.16	0.91–1.48	0.2267
History of cardiac disease (positive/negative)	0.95	0.80–1.12	0.5334
History of dyslipemia (positive/negative)	0.95	0.80–1.14	0.6096
History of malignancy (positive/negative)	1.02	0.76–1.37	0.8861
History of B/C hepatitis (positive/negative)	0.75	0.53–1.08	0.1206
Number of HLA-A-B-DR mismatches (>4/≤4)	1.46	1.18–1.81	0.0004
Anti-class I PRA (positive/negative)	1.07	0.87–1.31	0.5205
Anti-class II PRA (positive/negative)	1.29	1.04–1.59	0.0180
Induction therapy (depleting/non depleting)	0.65	0.54–0.77	<0.0001
Cold ischemia time (≥24 h/<24 h)	0.88	0.73–1.07	0.1984
Donor age (≥55 years/<55 years)	1.06	0.89–1.27	0.5027
Recipient/donor relationship (deceased donor/living donor)	0.92	0.73–1.15	0.4490

CI, confidence interval; BMI, body mass index; PRA, panel reactive antibody; HLA, human leukocyte antigen.

The cumulative probability of severe ARE at 1 and 12 months respectively were 2% and 5% for STR, and 1% and 2% for FTR (Figure 2-B). The univariate analysis showed that STR had a higher risk of severe ARE occurrence than FTR (p = 0.0040), but this significant result was not confirmed by the multivariate Cox model (Table 4, HR = 1.27, p = 0.4087). Severe ARE occurrence was also related to anti-class II PRA (HR = 2.26, p = 0.0027). Of note, recipients transplanted before 2005 (HR = 0.52, p = 0.0329) and recipients of an old donor graft (HR = 0.59, p = 0.3470) had a significantly lower risk of severe ARE occurrence.

**Table 4:** Multivariate Cox model analysis of severe acute rejection episode (severe ARE)-free time risk factors (N = 3103).

Variables	Hazard Ratio	95% CI	p
Graft rank (2/1)	1.27	0.72–2.21	0.4087
Transplantation period (<2005/≥2005)	0.52	0.28–0.95	0.0329
Recipient gender (male/female)	1.10	0.71–1.68	0.6762
Recipient age (≥55 years/<55 years)	1.52	0.93–2.48	0.0930
Causal nephropathy (recurrent/non recurrent)	0.97	0.63–1.51	0.9086
History of diabetes (positive/negative)	0.85	0.40–1.80	0.6769
History of hypertension (positive/negative)	1.41	0.78–2.54	0.2516
History of vascular disease (positive/negative)	0.99	0.51–1.91	0.9758
History of cardiac disease (positive/negative)	0.92	0.59–1.44	0.7299
History of dyslipemia (positive/negative)	0.81	0.49–1.33	0.4045
History of malignancy (positive/negative)	0.76	0.34–1.68	0.4952
History of B/C hepatitis (positive/negative)	0.51	0.19–1.41	0.1964
Number of HLA-A-B-DR mismatches (>4/≤4)	1.34	0.76–2.36	0.3051
Anti-class I PRA (positive/negative)	1.20	0.72–2.00	0.4911
Anti-class II PRA (positive/negative)	2.26	1.33–3.85	0.0027
Induction therapy (depleting/non depleting)	0.84	0.53–1.33	0.4545
Cold ischemia time (≥24 h/<24 h)	1.46	0.93–2.29	0.0997
Donor age (≥55 years/<55 years)	0.59	0.36–0.96	0.0347
Recipient/donor relationship (deceased donor/living donor)	0.93	0.46–1.87	0.8427

CI, confidence interval; BMI, body mass index; PRA, panel reactive antibody; HLA, human leukocyte antigen.

## Discussion

Based on an overview of the literature, the prognosis of STR compared to FTR is still unclear. As the demand for kidney transplants largely exceeds the supply, it is important to evaluate the excess risk related to STR and to identify patients with the worst chances of long-term outcome.

In 2003, Coupel et al. compared 233 STR to 1174 FTR and observed no difference in the 10-year survival [14], probably as STR were younger and had a higher level of HLA-matching than FTR. In 2008, Arnol et al. reported a similar 15-year survival between 81 STR compared to 427 FTR. They also found no differences in the occurrence of ARE between the two groups [15]. From a series of 26 deceased-donor STR versus 140 FTR analyzed in 2009, Gruber et al. also reported no differences in the 8-year survival nor in the occurrence or severity of ARE [5]. In the same year, Wang et al. compared the 5-year PGS of 65 deceased-donor STR versus 613 FTR and likewise, reported no difference [16]. Thus, from these former studies, it appears that STR have a long-term outcome similar to FTR. However, the interpretations of these studies are limited by several factors: the slight number of STR (low statistical power), the monocenter design, the number of adjustment covariates or the short follow-up period. Conversely, in 2007, Magee et al. [17] compared the 5-year graft survival of a large cohort of kidney recipients (more than 2000 STR versus more than 20000 FTR), from the Organ Procurement and Transplantation Network registry, and reported that even with adjustment for donor and recipient factors, the 5-year risk of graft failure remained significantly higher for repeat kidney transplant recipients (including second transplantations and more) than for FTR. Nevertheless, adjustment factors were limited and the follow-up was short. Moreover none of these studies evaluated the possible time-dependent effect of the graft rank, which is the central assumption of the proportional hazard Cox model.

In this paper, we used a specific methodology for an accurate comparison between FTR and STR, by taking into account all the possible confounding factors and modeling the time-dependent effect of the graft rank. To our knowledge, such an analysis has never been performed. Our results, based on recipients from a large multicenter cohort under similar recent immunosuppressive maintenance therapy, show that STR have a poorer long-term prognosis than FTR. We show for the first time that this risk is delayed and appears significant beyond four years of follow-up. This cut-off definitely does not correspond to a sudden modification of the graft failure risk. We should rather retain that the excess of risk of STR appears after few years of transplantation. This time-dependent association may be a major point as it was only after its introduction that we showed the significant excess risk of graft failure for STR: it may explain that the majority of the other papers did not demonstrate significant correlation between the survival of FTR and STR.

The difference in PGS could have been due to a higher frequency of ARE or severe ARE for STR than for FTR during the follow-up. However, we did not demonstrate such a difference in ARE, nor in severe ARE occurrences. For this last endpoint, we showed that STR tended to have a higher risk of severe ARE than FTR. The lack of statistical power (only 96 severe ARE were observed in the whole cohort) may explain why this finding did not reach statistical significance.

As always in observational studies, there are several limitations to this study. (i) The use of different techniques for PRA identification may introduce a bias, limited by the fact that STR and FTR were compared over the same period/center and by adjusting on the year of transplantation. (ii) It was not possible to include the causes of graft loss in our analyses (immunologic versus non-immunologic causes) since this the collection of this information has only recently been initiated. (iii) It was unfortunately not possible to adjust for the pretransplant duration of dialysis and the duration of first transplant survival, as only covariates common to FTR and STR can be taken into account in a Cox model. To overcome this difficulty, we adjusted for the comorbidities at transplantation. (iv) Adjustment for long-term immunosuppression regimens was not done, as it is more a reflection of a therapeutic adaptation to a clinical situation and it depends on the center, on the clinician and on the therapeutic advances. (v) A possible effect of the transplantation policy might introduce some bias that is overcome by the adjustment in the multivariate model and by the matched case-control design in the sub-analysis model. (vi) Our study also failed to eliminate the effects of some confounding factors such as medication compliance; as in every large-sized cohort, this information cannot be realistically collected. (vii) Delayed graft function (DGF) was not included as a covariate in the analysis as only pre- and per-transplant covariates were taken into account. However, an additional analysis including DGF did not provide new possible explanations for the different first and second transplant outcomes (data not shown). (vii) Although all ARE were biopsy-proven, a small number were classified using the most recent Banff criteria. It will take a few years before we are able to explore the possible link between biopsy-proven antibody-mediated ARE occurrence and a worse outcome. (viii) Finally, due to the long-term follow-up period, the information about preformed DSA was available for only a very small part of our cohort, although this covariate is suspected to be related to risk of graft failure.

In conclusion, this observational study on a large multicenter cohort confirmed other findings showing that STR have a lower patient-and-graft survival compared to FTR. However, this study eliminates some confounding factors from the current literature. The excess risk of graft failure for STR was delayed after several years post transplantation. This effect did not seem to be related to a higher frequency of ARE or severe ARE for second grafts. Regardless of the limitations of such an observational cohort; the current study supports the hypothesis of a higher propensity for STR to develop donor specific antibodies post-transplantation. These findings justify further expensive systematic and prospective monitoring of antibodies in both populations. Further investigations are still needed to understand the biological/immunological mechanisms underlying graft failure, to identify patients specifically at risk of graft failure and also to provide a strategy for improving outcome in STR. But in practice, physicians should not consider second and first kidney transplant recipients equally.
